# Contrasting Population Dynamics of *Haloxylon ammodendr* on and *Haloxylon persicum* Along a Groundwater-Depth Gradient

**DOI:** 10.3390/plants15213489

**Published:** 2026-11-08

**Authors:** Zhe Hao, Tao Lin, Shengchuan Tian, Lan Du, Yugang Wang, Bin Zhang, Xinjun Zheng

**Affiliations:** 1 Field Scientific Observatory for Soil and Water Processes and Ecological Security in Oasis of Tarim River Headwaters Area, Ministry of Natural Resources, Xinjiang Comprehensive Land Consolidation and Rehabilitation Center, Urumqi 830002, China; 2 Technology Innovation Center for Ecological Monitoring and Restoration of Desert-Oasis, Ministry of Natural Resources, Xinjiang Comprehensive Land Consolidation and Rehabilitation Center, Urumqi 830002, China; 3 State Key Laboratory of Ecological Safety and Sustainable Development in Arid Lands, Xinjiang Institute of Ecology and Geography, Chinese Academy of Sciences, Urumqi 830011, China; 4 University of Chinese Academy of Sciences, Beijing 100049, China; 5 College of Ecology and Environment, Xinjiang University, Urumqi 830046, China

**Keywords:** *Haloxylon ammodendron*, *Haloxylon persicum*, groundwater depth, population dynamics, population structure, Leslie matrix

## Abstract

Groundwater-table decline is an important environmental driver of desert vegetation degradation in arid regions, yet population structure and growth potential of co-dominant species along groundwater-depth gradients remain insufficiently compared. To examine differences in population dynamics between *Haloxylon ammodendron* and *H. persicum*, five sites with groundwater depths of 4, 7, 10, 13, and 16 m were selected along the southern margin of the Gurbantunggut Desert. Population stage and size-class structure, seedling survival, and reproductive characteristics were surveyed, and population dynamics were evaluated using life tables, population structural dynamic indices, demographic parameters, and Leslie matrix projections. Both species had abundant seedlings but relatively few non-current-year individuals, and seedling mortality approached 98%. The density of *H. ammodendron* individuals in stages III–IX was relatively similar across the 4–13 m sites but substantially lower at 16 m. In contrast, the density of *H. persicum* was lower across the 7–16 m sites than at 4 m. With increasing groundwater depth, the intrinsic rate of increase and finite rate of increase in *H. ammodendron* declined significantly, whereas its population doubling time increased significantly; none of the demographic parameters of *H. persicum* showed a significant relationship with groundwater depth. Under constant model parameters, Leslie matrix projections indicated that *H. ammodendron* populations generally declined at groundwater depths of 10–16 m, whereas *H. persicum* populations remained relatively stable or increased. The two species showed contrasting population patterns across the groundwater-depth gradient, but the spatial design does not establish groundwater decline as their sole cause, and differences in response strength between species require direct statistical testing. The projections describe conditional trajectories rather than observed temporal changes. The findings provide a basis for population maintenance of dominant desert shrubs and vegetation conservation under continued groundwater-table decline in the Gurbantunggut Desert.

## 1. Introduction

Arid regions account for approximately 41% of the global land surface and constitute an important component of terrestrial ecosystems [1]. Owing to low precipitation and high evaporative demand, production, domestic water use, and ecological water requirements in inland river basins of arid regions depend heavily on groundwater, forming typical groundwater-dependent ecosystems (GDEs) [2]. Groundwater represents a critical and relatively stable water source for vegetation in water-limited environments and can influence vegetation distribution, diversity, structure, productivity, and persistence, particularly during drought [2,3]. In recent years, increasing attention has been paid to changes in groundwater and the associated variation in soil salinity, both of which are closely related to the growth and distribution of *Haloxylon ammodendron* [4].

The southern margin of the Gurbantunggut Desert is a typical desert–oasis ecotone. In this region, the growth and distribution of *H. ammodendron* are jointly influenced by groundwater depth, groundwater quality, and soil salinity [4]. *Haloxylon ammodendron* and *H. persicum* are important dominant species of desert vegetation in this region and play key roles in wind erosion control, sand stabilization, biodiversity maintenance, and ecosystem stability. The two species differ in habitat preference and water-use characteristics. *H. persicum* mainly occurs on dune slopes and crests and shows greater tolerance to drought and high temperatures, whereas *H. ammodendron* is more commonly distributed in interdune lowlands and exhibits greater tolerance to salinity [5]. Stable-isotope studies have further shown that the two Species differ in their water sources and water-use strategies: *H. ammodendron* can utilize groundwater, whereas *H. persicum* relies more strongly on deep soil water [6,7]. Recent field studies have also demonstrated contrasting responses of the two species to groundwater decline, with *H. ammodendron* showing stronger reductions in growth and population performance under deeper groundwater conditions than *H. persicum* [8,9]. Comparative work on *H. ammodendron* and *Tamarix ramosissima* also shows that groundwater availability and prolonged summer drought can jointly influence water sources, gas exchange, and plant water status [10]. This provides regional physiological context, although it does not directly compare the two *Haloxylon* species.

Population structure and numerical dynamics reflect the current status and future trajectory of plant populations and provide an important basis for understanding plant–environment relationships [11,12,13]. Previous studies have examined the population structure, age composition, regeneration, and dynamic characteristics of *H. ammodendron* and *H. persicum* [8,14,15,16,17]. Recent work has further shown that groundwater depth can strongly constrain regeneration of *H. ammodendron*, whereas *H. persicum* exhibits greater tolerance and reproductive capacity under deeper groundwater conditions [8]. However, following groundwater-table decline, plants may continue to survive by using residual soil water and precipitation, resulting in delayed population responses. Consequently, short-term observations may not fully capture the effects of groundwater change on population dynamics [18]. Integrating seedling survival, the structure of non-current-year individuals, reproductive characteristics, and matrix-based population projections therefore provides a more comprehensive framework for comparing population maintenance processes of the two species along groundwater-depth gradients. These studies establish important physiological and regeneration responses, but the relationship between early seedling losses, the size structure of established individuals, and conditional population growth across the same groundwater-depth gradient remains insufficiently integrated. The present study links these components through site-level life tables, structural indices, demographic parameters, and matrix projections; its contribution is an integrated population-level comparison rather than a new test of physiological mechanisms.

In the desert–oasis ecotone of the Sangong River Basin, a natural clay barrier has maintained a relatively stable groundwater-depth gradient in the desert interior over recent decades, providing suitable conditions for comparing population characteristics across groundwater depths [18]. Accordingly, we selected five sites with groundwater depths of 4, 7, 10, 13, and 16 m along the southern margin of the Gurbantunggut Desert. We investigated the population structure, seedling survival, and reproductive characteristics of *H. ammodendron* and *H. persicum*, and compared their population dynamics along the groundwater-depth gradient using life tables, population structural dynamic indices, demographic parameters, and Leslie matrix projections. Specifically, we addressed the following questions: (1) How do the population structure and survival characteristics of the two *Haloxylon* species vary with groundwater depth? (2) How are demographic parameters associated with groundwater depth in each species? and (3) Under the specified model assumptions, do the projected population trajectories of the two species differ among groundwater depths? By identifying seedling establishment and the maintenance of established individuals as distinct components of population persistence, this comparison can inform groundwater-linked monitoring and habitat-specific conservation of dominant desert shrubs.

## 2. Materials and Methods

### 2.1. Study Area and Site Selection

**Figure 1:**
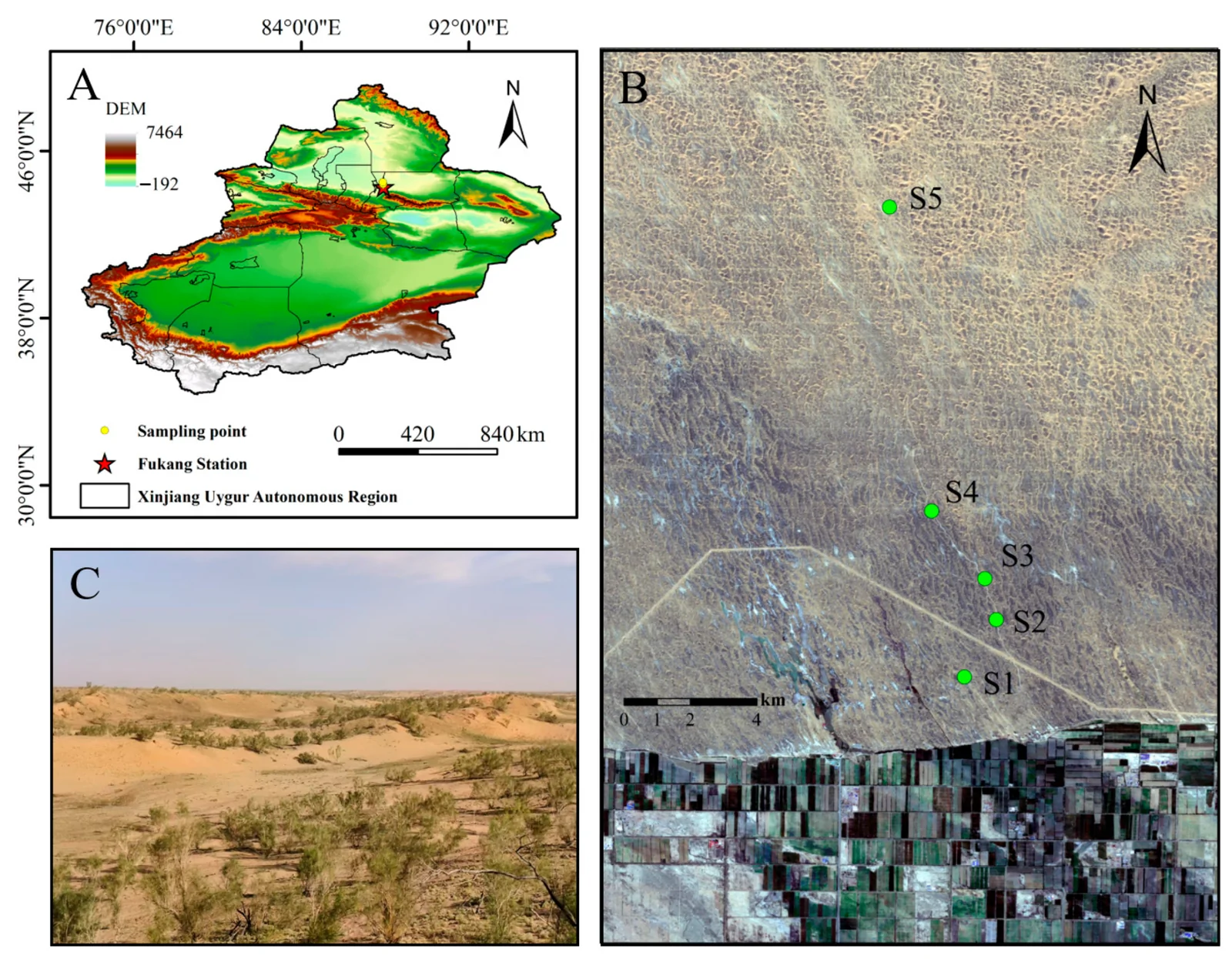
Location of the study area, distribution of sampling sites, and landscape of the study sites. (**A**) Geographical location and topographic background of the study area in Xinjiang; ★ indicates the Fukang Desert Ecosystem National Observation and Research Station, Chinese Academy of Sciences. (**B**) Remote-sensing image of the study area and distribution of sites S1–S5. (**C**) Typical landscape of the study area. Note: The base map was produced using the standard map from the Ministry of Natural Resources (Map Approval Number: GS(2020)4642), and no modifications were made to the base map boundaries.

The study area was located in the desert–oasis ecotone of the Sangong River Basin along the southern margin of the Gurbantunggut Desert (Figure 1). The region has a temperate continental climate, with annual precipitation of 70–150 mm, occurring mainly in spring and winter. Annual potential evaporation exceeds 2000 mm, stable snow cover occurs in winter, and maximum summer temperatures can exceed 40 °C [7]. The dominant shrubs include *Haloxylon ammodendron*, *H. persicum*, *Tamarix ramosissima*, and *Reaumuria soongarica*, together with various ephemeral plants and biological soil crusts. Site selection was based on the existing groundwater-monitoring well network, preliminary groundwater surveys, and established permanent plots in the study area. Preliminary survey information and real-time groundwater monitoring were used to identify five sites representing different positions along the local groundwater-depth gradient. The mean groundwater depths during the survey period at the selected sites (S1–S5) were 4, 7, 10, 13, and 16 m, respectively, based on real-time groundwater monitoring. This design allowed population characteristics of the two species to be compared across an existing 12 m groundwater-depth range using a space-for-time approach [19]. The five depths represent observed site conditions rather than experimentally imposed treatments or predetermined ecological thresholds.

### 2.2. Population Survey and Stage/Size-Class Classification

In 2021, four 100 m × 10 m belt transects were established for *H. ammodendron* and four for *H. persicum* at each groundwater-depth site, giving eight transects per site. Each transect was divided into ten contiguous 10 m × 10 m quadrats, resulting in 40 quadrats per species per site. To construct life tables and Leslie matrices from continuous size-class frequency distributions and improve the representation of population size structure through a larger sample, valid individual records from all quadrats within each site were pooled by species; transect-level spatial replication was not retained in these analyses. In June, individuals within the transects were counted and their basal stem diameters (BSD, cm) were recorded. Four 5 m × 5 m subplots were established to monitor the survival of newly emerged seedlings from April to October.

In October, whole-plant fruit production was estimated using an average standard-branch method. Branches judged to be intermediate in size (volume) and fruit abundance were selected to represent the average fruit-bearing condition of each plant. Fruits on the selected branches were counted, and their mean fruit count was calculated. Whole-plant fruit production was estimated by multiplying this mean by the estimated number of equivalent standard branches on the plant. This average standard-branch procedure was distinct from probability-based randomized branch sampling. Branch selection relied on observer judgement rather than known selection probabilities; consequently, this procedure did not provide a design-based estimate of sampling error.

### 2.3. Life-Table Construction

Individuals were first distinguished as current-year seedlings or non-current-year individuals. Current-year seedlings comprised two successive monitoring stages: stage I included seedlings emerging in spring, and stage II included those surviving into summer. These were successive states of the same seedling cohort, used to describe early seedling losses, rather than independent age groups. Because chronological ages were difficult to determine in the field, non-current-year individuals were classified by basal stem diameter (BSD), used as a size-based indicator of development rather than a direct measure of age. Seven size classes were defined: III, 0 < BSD ≤ 2 cm; IV, 2 < BSD ≤ 4 cm; V, 4 < BSD ≤ 6 cm; VI, 6 < BSD ≤ 8 cm; VII, 8 < BSD ≤ 10 cm; VIII, 10 < BSD ≤ 12 cm; and IX, BSD > 12 cm. Current-year seedlings were assigned to stages I and II irrespective of BSD, so class III contained only non-current-year individuals.

The life-table analysis incorporated stages I–II and size classes III–IX, combining monitored early seedling survival with the cross-sectional size distribution of non-current-year individuals.

Following the principles of static life-table construction [20], segmented smoothing was applied to stage-specific abundances according to Jiang to avoid theoretically negative mortality rates caused by environmental heterogeneity, sampling error, or fluctuations in the number of individuals among stages [21]. Let *a_x_* denote the observed number of individuals in stage x and *l_x_* the smoothed number of survivors in stage x. Life-table parameters were calculated as follows:

The number of deaths between stages *x* and *x* + 1 ($d_{x}$) was calculated as: $d_{x}=l_{x}-l_{x+1}$; The mortality rate ($q_{x}$) was calculated as: $q_{x}=\frac{d_{x}}{l_{x}}\times 100\%$; The mean number of individuals surviving between stages x and x + 1 ($L_{x}$) was calculated as: $L_{x}=\frac{\left(l_{x}+l_{x+1}\right)}{2}$; The cumulative number of survivors from stage x onward ($T_{x}$) was calculated as: $T_{x}=\sum_{x}^{\infty }L_{x}$; Life expectancy at stage x ($e_{x}$) was calculated as: $e_{x}=\frac{T_{x}}{l_{x}}$; The disappearance rate at stage x ($K_{x}$) was calculated as: $K_{x}=\ln l_{x}-\ln l_{x+1}$; The survival rate from stage x to stage x + 1 ($S_{x}$) was calculated as: $S_{x}=\frac{l_{x+1}}{l_{x}}$.

Here, x denotes population stage or size class and k denotes the maximum stage or size class. These parameters were used to characterize survival, mortality, and life expectancy of the two Haloxylon populations across groundwater depths.

### 2.4. Calculation of Population Structural Dynamic Indices

Following the quantitative method for plant population structural dynamics proposed by Chen [22], changes in the number of individuals between adjacent stages or size classes were quantified using the adjacent-stage dynamic index ($V_{n}$), the overall population dynamic index without external disturbance ($V_{p}$), the index corrected for completely random disturbance ($V_{p}^{\prime }$), and the index under non-completely random disturbance ($V_{p}^{\prime \prime }$). To avoid confusion with the survival rate $S_{x}$ defined in Section 2.3, $N_{n}$ was used here to denote the number of individuals in stage or size class *n*.

The dynamic index between adjacent stages *n* and ${n+1\;V}_{n}$ was calculated as:

$$V_{n}=\frac{\left(N_{n}\;-\;N_{n+1}\right)}{max\left(N_{n},N_{n+1}\right)}$$

Values of $V_{n}$ > 0, $V_{n}$ = 0, and $V_{n}$ < 0 indicate increasing, stable, and declining numerical relationships between adjacent stages, respectively.

The overall population dynamic index in the absence of external disturbance ($V_{p}$) was calculated as:

$$V_{p}=\frac{\sum_{n-1}^{k-1}N_{n}V_{n}}{\sum_{n-1}^{k-1}N_{n}}$$

where *k* is the total number of population stages or size classes. Values of $V_{p}\;$> 0, $V_{p}$ = 0, and $V_{p}$ < 0 indicate an overall increasing, stable, and declining population structure, respectively.

Under completely random external disturbance, the maximum disturbance-risk probability ($P_{max}$) was calculated as:

$$P_{max}=\frac{1}{k\cdot min\left(N_{1},N_{2},\ldots ,N_{k}\right)}$$

The population dynamic index corrected for completely random disturbance ($V_{p}^{\prime }$) was calculated as:

$$V_{p}^{\prime }=P_{max}V_{p}$$

$$V_{p}^{\prime }=\frac{\sum_{n-1}^{k-1}N_{n}V_{n}}{k\cdot min\left(N_{1},N_{2},\ldots ,N_{k}\right)\cdot \sum_{n}^{k-1}N_{n}}$$

To account for differences in disturbance intensity among stages, the relative mortality of stage *n* ($D_{n}$) was calculated as:

$$D_{n}=\frac{\left(N_{n}-N_{n+1}\right)}{N_{n}}$$

The relative disturbance weight of stage *n* ($P_{n}$) was calculated as:

$$P_{n}=\frac{D_{n}}{\sum_{n=1}^{k-1}D_{n}}$$

The maximum risk probability under non-completely random disturbance ($P_{max}^{\prime }$) was calculated as:

$$P_{max}^{\prime }=max\left(\frac{P_{1}}{N_{1}},\frac{P_{2}}{N_{2}},\ldots ,\frac{P_{k}}{N_{k}}\right)$$

Accordingly, the population dynamic index under non-completely random disturbance ($V_{p}^{\prime \prime \;}$) was calculated as:

$$V_{p}^{\prime \prime }=P_{max}^{\prime }V_{p}$$

$$V_{p}^{\prime \prime }=\frac{max\left(\frac{P_{1}}{N_{1}},\frac{P_{2}}{N_{2}},\ldots ,\frac{P_{k}}{N_{k}}\right)\cdot \sum_{n=1}^{k-1}N_{n}V_{n}}{\sum_{n=1}^{k-1}N_{n}}$$

All dynamic indices ($V_{n}$, $V_{p}$, $V_{p}^{\prime }$, and $V_{p}^{\prime \prime \;}$) were expressed as proportions rather than percentages. Values of $V_{p}^{\prime }$ and $V_{p}^{\prime \prime \;}$ closer to zero indicate weaker population growth tendencies after accounting for external disturbance.

### 2.5. Survival-Curve Fitting

Curves were constructed using the natural logarithm of the number of surviving individuals as the dependent variable and stage or size-class number as the independent variable. Following Hett and Loucks [23], power and exponential functions were fitted empirically, and model performance was evaluated using the coefficient of determination (*R*^2^), *F*-statistics, and significance levels. Because the horizontal axis combined seasonal seedling stages with basal-diameter classes, these curves were used primarily to describe population numerical structure; their correspondence with classical age-based survivorship curves [24] was interpreted with consideration of the observation period and fitted variables.

### 2.6. Calculation of Demographic Parameters

The seed production of individuals in different stages or size classes was measured using the standard-branch method, and fecundity schedules were constructed in combination with stage-specific survival rates from the life table. Let *x* denote stage or size class, $S_{x}$ the survival rate of individuals in stage *x*, and m_x_ the mean number of seeds produced per individual in stage *x*. Following Xiao et al. [25], the net reproductive rate, intrinsic rate of increase, finite rate of increase, mean generation time, and population doubling time were calculated as follows.

The net reproductive rate ($R_{0}$) was calculated as:

$$R_{0}=\sum_{x}S_{x}m_{x}$$

The intrinsic rate of increase ($r_{m}$) was calculated as:

$$r_{m}=\frac{\ln R_{0}}{T}$$

The finite rate of increase (λ) was calculated as:

$$\lambda =e^{r_{m}}$$

The mean generation time (*T*) was calculated as:

$$T=\frac{\sum_{x}{xS}_{x}m_{x}}{\sum_{x}S_{x}m_{x}}$$

Because $R_{0}=\sum_{x}S_{x}m_{x}$, mean generation time can also be written as:

$$T=\frac{\sum_{x}{xS}_{x}m_{x}}{R_{0}}$$

Population doubling time (*t*) was calculated as:

$$t=\frac{\ln\left(2\right)}{r_{m}}$$

Here, *R*_0_ represents the number of effective offspring produced per individual over its lifetime; $r_{m}$ represents the potential population growth rate under the specified life-table parameters; *λ* represents the finite multiplication rate per unit time; *T* represents mean generation time; and t represents the theoretical time required for the population to double in size. These parameters were used to compare reproductive capacity and potential population growth of *H. ammodendron* and *H. persicum* across groundwater depths.

### 2.7. Leslie Matrix Model

Leslie matrix models were constructed using class-specific population size, survival, and fecundity [26]. The projections included only non-current-year individuals in size classes III–IX; seedling stages I and II were excluded from the projected population vector. The seven classes were ordered by increasing BSD. Population sizes were projected over five model time steps at each site, with the specified demographic parameters held constant.

### 2.8. Data Analysis

Statistical analyses were performed using IBM SPSS Statistics 21.0. Origin 8.5 was used for curve fitting and figure preparation, and Microsoft Excel 2010 was used for basic data processing and calculations. Pearson correlation analysis was used to test relationships between groundwater depth and population structural dynamic indices and demographic parameters. Statistical significance was evaluated at *p* < 0.05. Analyses were conducted separately for each species using five site-level observations (*n* = 5). Individual plants, transects, quadrats, and repeated seedling observations were not counted as additional groundwater-depth replicates. Each groundwater-depth level was represented by one site; consequently, groundwater-depth associations could not be separated fully from other site-level differences.

## 3. Results

### 3.1. Population Structure of Haloxylon ammodendron and H. persicum Across Groundwater Depths

**Figure 2:**
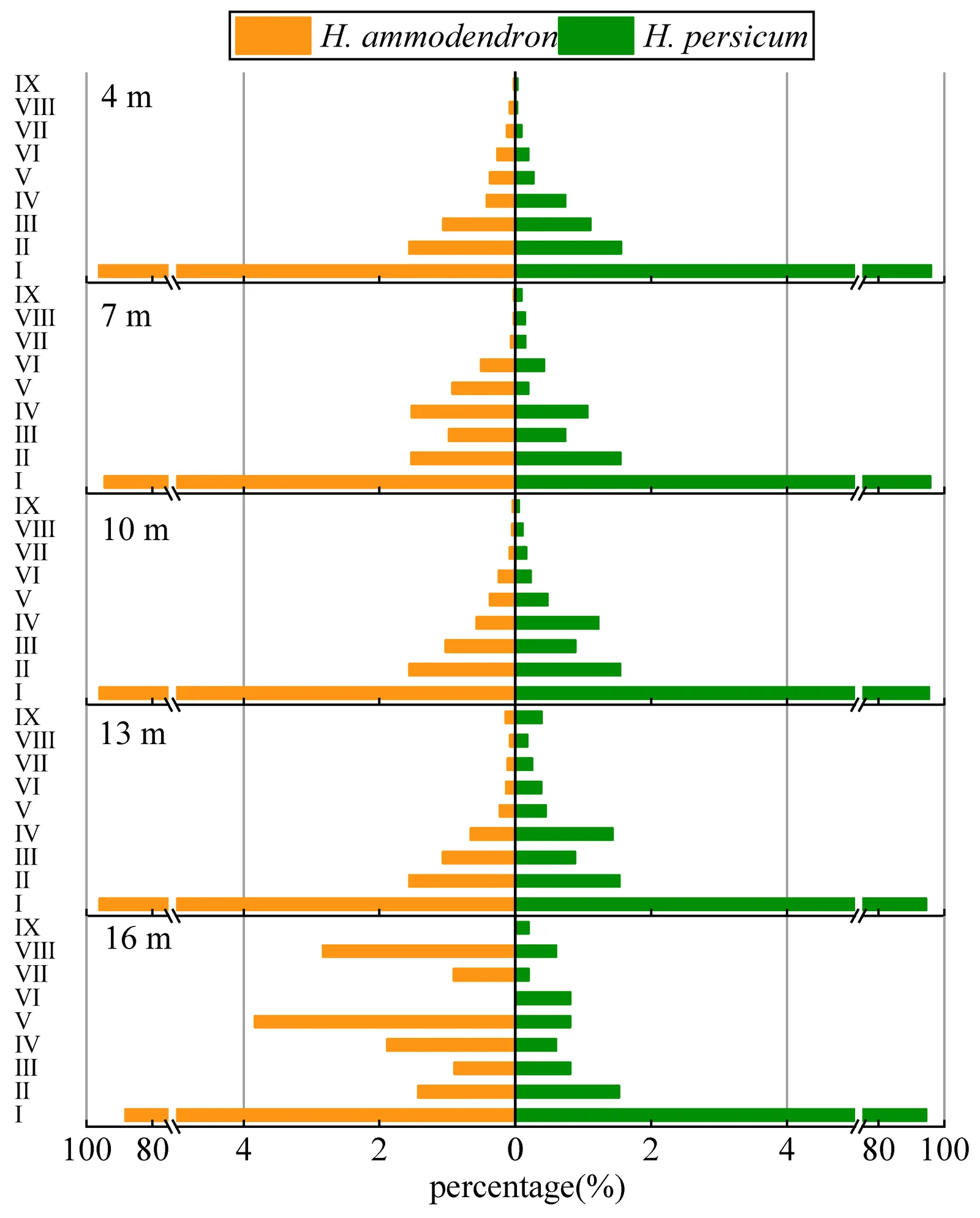
Population stage and size-class structure of *H. ammodendron* and *H. persicum* across groundwater depths. Stages I and II represent spring-emerged current-year seedlings and those surviving into summer, respectively. Classes III–IX represent non-current-year individuals classified by basal stem diameter (BSD): III, 0 < BSD ≤ 2 cm; IV, 2 < BSD ≤ 4 cm; V, 4 < BSD ≤ 6 cm; VI, 6 < BSD ≤ 8 cm; VII, 8 < BSD ≤ 10 cm; VIII, 10 < BSD ≤ 12 cm; and IX, BSD > 12 cm.

Both species exhibited an L-shaped stage and size-class distribution, with abundant current-year seedlings and relatively few individuals in the larger size classes (Figure 2). The density of non-current-year *H. ammodendron* individuals changed relatively little across sites with groundwater depths of 4–13 m but was substantially lower at 16 m, where classes VI and IX were absent.

In contrast, *H. persicum* density was much lower at 7 m than at 4 m and remained comparatively low across the 7–16 m sites. *H. persicum* had a higher density of non-current-year individuals than *H. ammodendron* at 4 and 16 m, whereas the opposite pattern occurred at 7–13 m.

### 3.2. Population Structural Dynamic Indices

**Figure 3:**
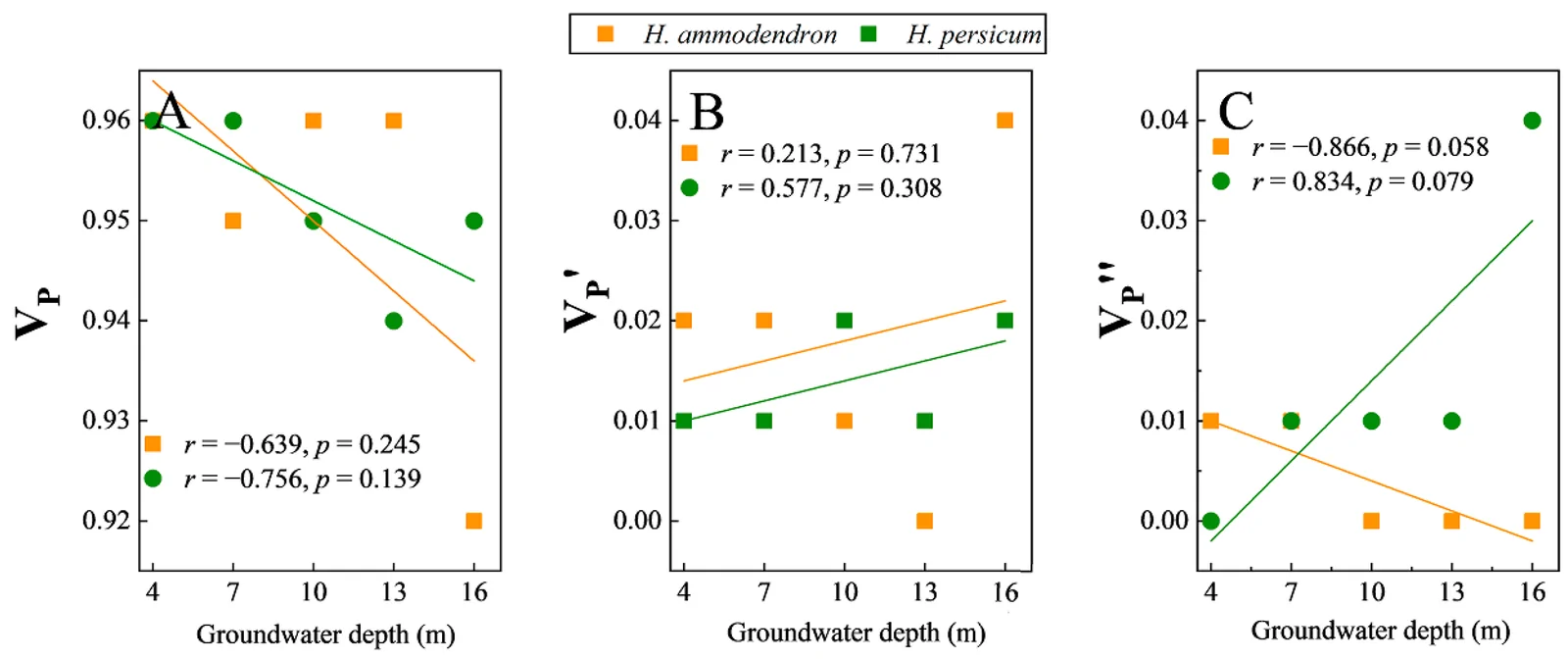
Relationships between groundwater depth and population structural dynamic indices of *H. ammodendron* and *H. persicum*. (**A**) Population dynamic index without external disturbance ($V_{p}$); (**B**) population dynamic index under completely random disturbance ($V_{p}^{\prime }$); and (**C**) population dynamic index under non-completely random disturbance ($V_{p}^{\prime \prime }$). Orange squares and green circles represent *H. ammodendron* and *H. persicum*, respectively. Lines indicate linear fits. All indices are dimensionless. The *r* and *p* values indicate Pearson’s correlation coefficients and associated *p*-values, respectively.

Population structural dynamic indices were non-negative for both species across groundwater depths (Figure 3). Indices calculated without external disturbance were close to 1 and exceeded those calculated under completely random and non-completely random disturbance.

None of the indices was significantly correlated with groundwater depth in either species (all *p* > 0.05). Under non-completely random disturbance, the index showed a negative association with groundwater depth in H. *ammodendron* and a positive association in *H. persicum*, although neither relationship was significant.

### 3.3. Survivorship-Curve Fitting

Power and exponential functions were fitted to the survivorship curves of *H. ammodendron* and *H. persicum* across groundwater depths, and all fitted models were highly significant (*p* < 0.01) (Table 1). The coefficients of determination *R^2^* ranged from 0.8347 to 0.9547 for *H. ammodendron* and from 0.8337 to 0.9490 for *H. persicum*.

For *H. ammodendron* at the 7 m groundwater depth, the survivorship curve was better fitted by an exponential function (*R*^2^ = 0.8347) and was classified as Deevey type I. At the remaining four groundwater depths, the survivorship curves of *H. ammodendron* were better fitted by power functions and were classified as Deevey type III. For *H. persicum*, power functions provided the better fit at all five groundwater depths, and the survivorship curves were classified as Deevey type III.

### 3.4. Demographic Parameters

**Figure 4:**
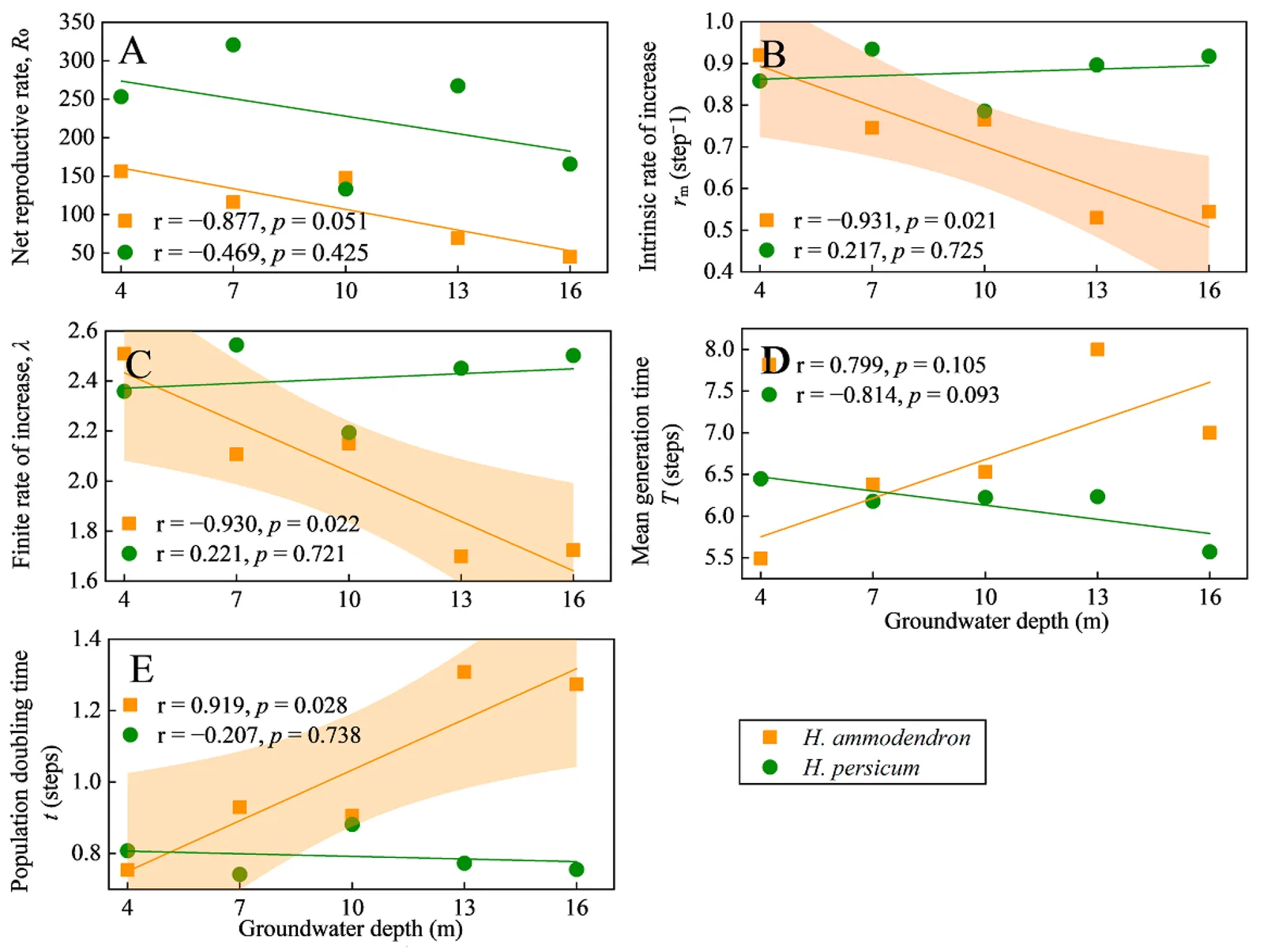
Relationships between groundwater depth and demographic parameters of *Haloxylon ammodendron* and *H. persicum*. (**A**) Net reproductive rate (*R*_0_); (**B**) intrinsic rate of increase (*r*_m_); (**C**) finite rate of increase (*λ*); (**D**) mean generation time (*T*); and (**E**) population doubling time (*t*). Orange squares and green circles represent *H. ammodendron* and *H. persicum*, respectively. Lines indicate linear fits. *R*_0_ and *λ* are dimensionless; *r*_m_ is expressed in step^−1^, and *T* and t in model steps. The *r* and *p* values indicate Pearson’s correlation coefficients and associated *p*-values, respectively.

In *H. ammodendron*, the intrinsic rate of increase and finite rate of increase declined significantly with increasing groundwater depth (Pearson *r* = −0.931, *p* = 0.021 and *r* = −0.930, *p* = 0.022, respectively), whereas population doubling time increased significantly (*r* = 0.919, *p* = 0.028; Figure 4). Net reproductive rate showed a negative association that did not reach statistical significance (*r* = −0.877, *p* = 0.051), and mean generation time was not significantly related to groundwater depth (*p* = 0.105).

None of the demographic parameters of *H. persicum* showed a significant relationship with groundwater depth (all *p* > 0.05).

### 3.5. Projected Population Dynamics Based on Leslie Matrices

**Figure 5:**
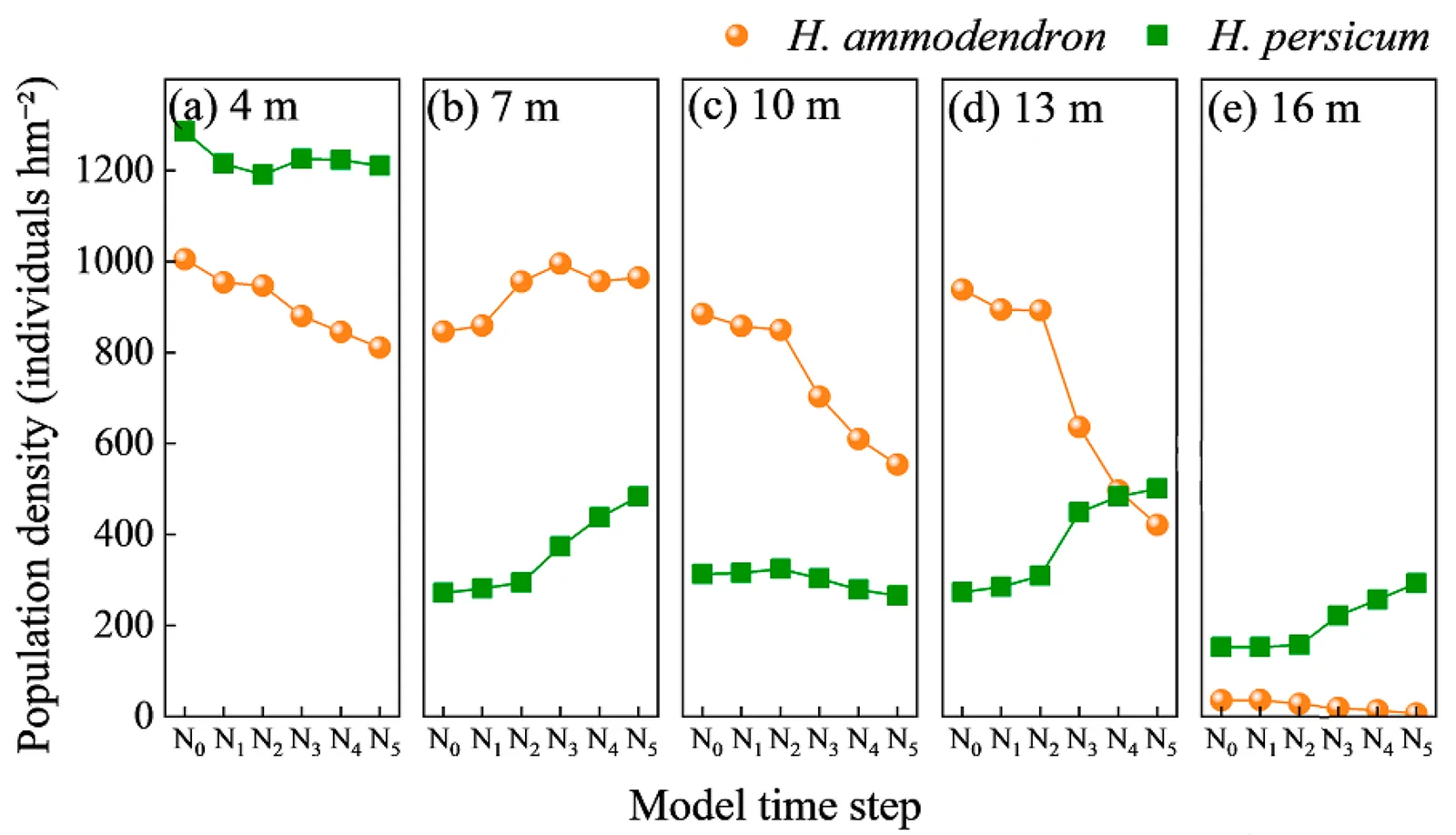
Projected densities of *H. ammodendron* and *H. persicum* over five model time steps along a groundwater-depth gradient.

Under constant model parameters, projected trajectories of individuals in classes III–IX differed between species and sites over five model time steps (Figure 5).

At 4 m, *H. ammodendron* declined, whereas *H. persicum* initially declined and then stabilized. Both species increased slightly at 7 m. At 10–16 m, *H. ammodendron* declined, whereas *H. persicum* remained relatively stable at 10 m and increased at 13 and 16 m.

## 4. Discussion

### 4.1. Population Structure and Survivorship Characteristics of the Two Haloxylon Species Along the Groundwater-Depth Gradient

Across groundwater depths, populations of both *H. ammodendron* and *H. persicum* showed an L-shaped structure characterized by a high proportion of juvenile individuals and relatively few individuals in the middle and larger size classes. The monitored seedling cohort was abundant in spring, but seedling mortality approached 98%, indicating that high seedling abundance did not translate into a corresponding number of established individuals in later classes. Previous studies have likewise reported high mortality during the seedling stage of *H. ammodendron*, suggesting that seedling establishment is a critical phase in population regeneration [27,28]. Recent research on the same two *Haloxylon* species has also emphasized the importance of population age structure and regeneration capacity for long-term population persistence under groundwater decline [8]. In the present study, the fitted abundance curves showed a pronounced reduction between early seedling stages and later size classes, consistent with the observed stage and size-class structures.

Seedlings of both species exhibited high mortality across groundwater depths. Because seedlings of *H. ammodendron* and *H. persicum* have relatively shallow root systems and primarily depend on near-surface soil water during early establishment, their survival may be more strongly affected by spring soil-moisture conditions than by direct access to groundwater. In the study region, rapid warming after snowmelt can lead to a rapid decline in surface-soil moisture. At the same time, ephemeral plants and biological soil crusts also use water from shallow soil layers, which may further increase water limitation during seedling establishment [29,30,31]. Studies have also examined seedling emergence of the two species under reciprocal exchange of habitat soil substrates and different sand-burial depths [32].

The observed result was a contrast in the distribution of established individuals across sites: *H. ammodendron* density was relatively similar at groundwater depths of 4–13 m but substantially lower at 16 m, whereas *H. persicum* density was lower across the 7–16 m sites than at 4 m.

Here, groundwater denotes water in the saturated zone below the local water table, distinguished from soil water in the unsaturated zone above it. Describing a species as groundwater dependent refers to its contribution to plant water uptake and does not imply that all individuals maintain direct root contact with the water table at every site. Isotope evidence indicates that both species can use groundwater, but *H. ammodendron* is more groundwater dependent and the contribution varies seasonally [6,7]. *H. ammodendron* has a deep, vertically developed root system with lateral roots; excavation studies show that rooting depth and root distribution vary with soil texture, with deeper rooting in sandy than in heavier soils [33]. *H. persicum* also develops belowground systems capable of accessing deep water, and its biomass allocation varies along groundwater-depth gradients [34]. These studies do not establish a fixed species-specific rooting depth or demonstrate root access to the 16 m water table in our plots.

Published comparative data also show why water-potential differences should be interpreted in their environmental context. Zhang et al. [35] measured predawn and midday water potentials of assimilative branches in both species along dune-to-lowland transects. They reported broadly similar species-level water potentials, while midday water potential increased with relative elevation across individuals; *H. ammodendron* showed lower hydraulic efficiency but greater hydraulic safety and tissue water storage. Thus, these observations do not support a universal ranking of one species as having lower water potential under all conditions. Previous groundwater-gradient studies nevertheless found physiological responses associated with greater groundwater dependence in *H. ammodendron* [7,36]. Root distributions, plant water sources, and water potentials were not measured in the present study. These mechanisms are therefore literature-supported hypotheses rather than processes demonstrated by our population observations.

In addition, both species exhibited more than one mortality peak during later developmental stages, indicating that mortality was not confined to the seedling stage. Previous studies have shown that drought resistance and recovery capacity differ among developmental stages [37]. Because root distribution, hydraulic traits, and carbon balance were not directly measured in this study, the physiological mechanisms underlying mortality peaks in older individuals remain to be clarified.

### 4.2. Contrasting Responses of Population Dynamics and Reproductive Parameters

Population structural dynamic indices remained non-negative for both *H. ammodendron* and *H. persicum* across groundwater depths, but the indices calculated under random disturbance were markedly lower than those under no disturbance. Under the no-disturbance condition, both species showed relatively strong population growth tendencies, whereas growth was reduced under random or non-completely random disturbance. Similar structural-dynamic patterns have been reported for *H. ammodendron* and *H. persicum* populations in previous studies [14,15,16].

The large number of seedlings in both species should, however, be interpreted together with their very high seedling mortality. Evaluating population growth solely on the basis of juvenile abundance may overestimate actual regeneration potential, because the numerical dominance of seedlings did not persist into later classes. Population structural dynamics should therefore be interpreted in conjunction with seedling survival and the abundance of established non-current-year individuals. This interpretation is also consistent with recent evidence that population persistence of *H. ammodendron* depends strongly on regeneration success and maintenance of a balanced age structure under increasing groundwater depth [8].

The demographic analyses showed different patterns of association with groundwater depth in the two species. In *H. ammodendron*, the intrinsic rate of increase and finite rate of increase were negatively correlated with groundwater depth, whereas population doubling time was positively correlated. None of the corresponding relationships was significant in *H. persicum*. These patterns are consistent with previous evidence on regeneration constraints in *H. ammodendron* [8]. However, a significant relationship in one species and a non-significant relationship in the other do not, by themselves, demonstrate a significant difference between species. A direct species-by-groundwater-depth interaction test would be needed to evaluate differences in response strength. Because each groundwater-depth level was represented by one site, these relationships cannot be attributed exclusively to groundwater depth.

### 4.3. Conditional Population Projections Across Groundwater Depths and Their Ecological Implications

Under constant model parameters, the Leslie matrix projections indicated declining *H. ammodendron* populations at groundwater depths of 10–16 m, whereas *H. persicum* remained relatively stable or increased. These trajectories are conditional model outputs rather than observed temporal changes. They describe the consequences of maintaining the specified transition and fecundity parameters over successive model steps; they do not simulate the effects of a changing groundwater table. Their realization depends on whether demographic rates remain comparable to those represented in the models. These projected trajectories were broadly consistent with the observed patterns in population density and demographic parameters. They are also consistent with recent empirical evidence showing greater constraints on the survival, growth, and regeneration of *H. ammodendron* under deeper groundwater conditions. Mu et al. [8] reported that a groundwater depth of approximately 15 m represented a strong survival and regeneration constraint for *H. ammodendron*, whereas the same limitation was not observed for *H. persicum*. Similarly, Wu et al. [9] found that continued groundwater decline had stronger negative effects on *H. ammodendron* than on *H. persicum*, and that *H. persicum* became relatively more dominant under groundwater depths exceeding approximately 12 m. The agreement in response direction with previous studies provides ecological context for the projections, but does not independently validate the projected trajectories or establish the mechanisms responsible for them.

*Haloxylon ammodendron* typically occurs in interdune lowlands, whereas *H. persicum* is more common on dune slopes and crests, reflecting differences in water use and environmental tolerance. Previous studies have shown that *H. persicum* performs better than *H. ammodendron* under relatively water-limited and nutrient-poor conditions, whereas *H. ammodendron* has advantages under higher soil salinity and greater nitrogen and phosphorus availability [5,38]. Physiological evidence further indicates that *H. ammodendron* can adjust hydraulic traits and osmotic regulation as groundwater depth increases, but these adjustments do not fully compensate for reduced water availability, because growth still declines under deeper groundwater conditions [36]. These species-specific differences in water acquisition and stress tolerance provide possible ecological explanations for the observed site-level patterns, rather than evidence that these processes caused the population differences.

Regional evidence links the distribution and growth of *H. ammodendron* to groundwater depth, groundwater quality, and soil salt accumulation [4], while reciprocal transplant experiments demonstrate contrasting habitat performance of the two *Haloxylon* species [5]. Groundwater depth can affect the potential for upward salt transport, but the direction and magnitude of salt accumulation also depend on groundwater mineralization, soil texture, and evaporative conditions. Neither soil salinity nor groundwater mineralization was measured across our five sites. We therefore cannot establish a site-level relationship between salinity and groundwater depth or separate their contributions to the observed population patterns.

The results identify seedling establishment and the maintenance of established individuals as priorities for monitoring both species. Conservation planning should combine groundwater monitoring with repeated seedling censuses, measurements of soil moisture and salinity, and tracking of tagged non-current-year individuals. The five sampled depths do not define management thresholds, and the projections should not be used to prescribe species replacement. Long-term monitoring across replicated sites is needed to evaluate population trajectories and distinguish groundwater associations from other environmental differences.

## 5. Conclusions

(1) Both *H. ammodendron* and *H. persicum* populations were characterized by a high proportion of juveniles and high seedling mortality, indicating that the transition from seedlings to established later-stage individuals is a key component of population maintenance.
(2) The two species responded differently to increasing groundwater depth. Under deeper-groundwater conditions, *H. ammodendron* showed stronger declines in population density and demographic growth potential, whereas changes in *H. persicum* were comparatively weaker.
(3) Leslie matrix projections indicated that *H. ammodendron* populations generally declined under deeper-groundwater conditions, whereas *H. persicum* populations tended to remain stable or increase, resulting in divergent projected population dynamics between the two species.

## Figures and Tables

**Table 1:** Fitted models for population abundance curves of *H. ammodendron* and *H. persicum* along a groundwater-depth gradient.

Population	Groundwater Depth (m)	Fitted Curve	*R* ^2^	*F*
*H. ammodendron*	4	y = 10.506 × x^−0.5304^	0.9199	311.754
	7	y = 10.900 × e^−0.1871x^	0.8347	111.143
	10	y = 10.393 × x^−0.5457^	0.9445	435.448
	13	y = 10.015 × x^−0.4702^	0.9547	759.324
	16	y = 5.660 × x^−0.8196^	0.9034	109.541
*H. persicum*	4	y = 10.770 × x^−0.5306^	0.9397	418.440
	7	y = 8.824 × x^−0.5798^	0.9440	402.136
	10	y = 9.100 × x^−0.5989^	0.9490	407.145
	13	y = 8.154 × x^−0.4932^	0.8756	246.988
	16	y = 7.375 × x^−0.5499^	0.8337	143.007

## Data Availability

The data presented in this study are available on request from the corresponding author due to [privacy/legal/ethical] restrictions.

## References

[1] Berdugo M., Kéfi S., Soliveres S., Maestre F.T. (2017). Plant spatial patterns identify alternative ecosystem multifunctionality states in global drylands. Nat. Ecol. Evol..

[2] Huang F., Zhang Y., Zhang D., Chen X. (2019). Environmental groundwater depth for groundwater-dependent terrestrial ecosystems in arid/semiarid regions: A review. Int. J. Environ. Res. Public Health.

[3] Glanville K., Sheldon F., Butler D., Capon S. (2023). Effects and significance of groundwater for vegetation: A systematic review. Sci. Total Environ..

[4] Sun L., Liu T. (2016). Response of *Haloxylon ammodendron* (C. A. Mey.) to underground water quality, depth and soil salt deposition in Gurbantunggut Desert, West China. Pak. J. Bot..

[5] Xu G.-Q., Yu D.-D., Li Y. (2017). Patterns of biomass allocation in *Haloxylon persicum* woodlands and their understory herbaceous layer along a groundwater depth gradient. For. Ecol. Manag..

[6] Dai Y., Zheng X.J., Tang L.S., Li Y. (2015). Stable oxygen isotopes reveal distinct water use patterns of two *Haloxylon* species in the Gurbantunggut Desert. Plant Soil.

[7] Wu X., Zheng X.-J., Li Y., Xu G.-Q. (2019). Varying responses of two *Haloxylon* species to extreme drought and groundwater depth. Environ. Exp. Bot..

[8] Mu X.-H., Du L., Zheng X.-J., Tang L.-S., Ma J., Li Y. (2024). Population regeneration of two *Haloxylon* species in Central Asian deserts as affected by groundwater depth. Environ. Exp. Bot..

[9] Wu X., Li Y., Xu G.-Q. (2024). Response of two dominant woody species to groundwater depth at the transition zone between desert and oasis. Ecol. Indic..

[10] Tiemuerbieke B., Ma J.Y., Sun W. (2024). Differential eco-physiological performance to declining groundwater depth in Central Asian C3 and C4 shrubs in the Gurbantunggut Desert. Front. Plant Sci..

[11] Nathan R., Muller-Landau H.C. (2000). Spatial patterns of seed dispersal, their determinants and consequences for recruitment. Trends Ecol. Evol..

[12] Rees M., Condit R., Crawley M., Pacala S., Tilman D. (2001). Long-term studies of vegetation dynamics. Science.

[13] Nanami S., Kawaguchi H., Tateno R., Li C.H., Katagiri S. (2004). Sprouting traits and population structure of co-occurring *Castanopsis* species in an evergreen broad-leaved forest in southern China. Ecol. Res..

[14] Lv C., Zhang X., Liu G. (2014). Population characteristics of *Haloxylon ammodendron* (C. A. Mey.) Bunge in Gurbantunggut Desert, China. Pak. J. Bot..

[15] Cai X., Wu J. (2016). Characteristics and dynamics analysis of *Haloxylon persicum* populations in the Nature Reserve of Gan Jia Lake. J. Arid Land Resour. Environ..

[16] Li G., Zhang D., Zhang Z., Hu Y., Huang L., Lu L. (2021). Population dynamics of main sand-fixing shrubs in the Gurbantunggut Desert. J. Desert Res..

[17] Liu Y.X., Zeng Y., Yang Y.H., Wang N., Liang Y.J. (2022). Competition, spatial pattern, and regeneration of *Haloxylon ammodendron* and *Haloxylon persicum* communities in the Gurbantunggut Desert, Northwest China. J. Arid Land.

[18] Li Y., Zheng X., Wang Y., Xu G., Liu R. (2021). Experiment and simulation platform for oasis-desert symbiotic relationship (ODP). Bull. Chin. Acad. Sci..

[19] Blois J.L., Williams J.W., Fitzpatrick M.C., Jackson S.T., Ferrier S. (2013). Space can substitute for time in predicting climate-change effects on biodiversity. Proc. Natl. Acad. Sci. USA.

[20] Xie T., Su P., Zhou Z., Zhang H., Li S. (2014). Structure and dynamic characteristics of *Calligonum mongolicum* population in the desert–oasis ecotone. Acta Ecol. Sin..

[21] Jiang H. (1992). Population Ecology of Spruce.

[22] Chen X. (1998). A study on the method of quantitative analysis for plant population and community structural dynamics. Acta Ecol. Sin..

[23] Hett J.M., Loucks O.L. (1976). Age structure models of balsam fir and eastern hemlock. J. Ecol..

[24] Deevey E.S. (1947). Life tables for natural populations of animals. Q. Rev. Biol..

[25] Xiao Y., He P., Li X., Deng H. (2004). Study on numeric dynamics of natural populations of the endangered species *Disanthus cercidifolius* var. *longipes*. Acta Phytoecol. Sin..

[26] Zang R., Zhu N., Li J. (1994). Study on the population ecology of *Acanthopanax senticosus* in natural secondary forest communities (V): A Leslie matrix model for predicting the growth of the population. J. Jilin For. Inst..

[27] Liu G., Li J., Wang J., Wang J., Li J., Wang X. (2010). Analysis on dynamic life table of natural *Haloxylon ammodendron* at seedling stage in the southeastern marginal zone of the Junggar Basin. Arid Zone Res..

[28] Wang G., Zhao W. (2015). Effects of sand-burying on seed germination and seedling growth of *Haloxylon ammodendron*. J. Desert Res..

[29] Tian Y., Li J., Zhao Y. (2010). Relationship between *Haloxylon ammodendron* seedling mortality and water content in soil and atmosphere. J. Desert Res..

[30] Zhao L., Li W.J., Yang G., Yan K., He X.L., Li F.D., Gao Y.L., Tian L.J. (2021). Moisture, temperature, and salinity of a typical desert plant (*Haloxylon ammodendron*) in an arid oasis of northwest China. Sustainability.

[31] Li S., Bowker M.A., Xiao B. (2022). Biocrust impacts on dryland soil water balance: A path toward the whole picture. Glob. Change Biol..

[32] Li H., Li Y., Fan L. (2011). Responses of seedling emergence of two *Haloxylon* species to reciprocal exchange of habitat soil substrates and sand-burial depth. Arid Zone Res..

[33] Xu G.-Q., Li Y. (2008). Rooting depth and leaf hydraulic conductance in the xeric tree *Haloxyolon ammodendron* growing at sites of contrasting soil texture. Funct. Plant Biol..

[34] Xu G.Q., Yu D.D., Xie J.B., Tang L.S., Li Y. (2014). What makes *Haloxylon persicum* grow on sand dunes while *H. ammodendron* grows on interdune lowlands: A proof from reciprocal transplant experiments. J. Arid Land.

[35] Zhang C., Hao G.Y., Duan C.Y., Zhang J., Yin B.F., Zhang Y.M. (2026). Divergence in plant water relations mediates the clear niche differentiation of two congeneric desert shrub species. Plant Divers..

[36] Xu G.-Q., Mi X.-J., Ma J., Ma J., Tang L.-S. (2022). Impact of groundwater depth on hydraulic performance and growth of *Haloxylon ammodendron* in a desert region of Central Asia. Ecohydrology.

[37] Xu G.-Q., McDowell N.G., Li Y. (2016). A possible link between life and death of a xeric tree in desert. J. Plant Physiol..

[38] Xie J., Peng L., Li Y., Li Y. (2018). Competitive ability of seedlings of two *Haloxylon* species along soil nitrogen and phosphorus gradients. Arid Land Geogr..

